# Evaluation of vaccine immunity against tetanus, hepatitis B, measles, mumps, rubella and varicella, in migrant adolescents aged 13 to 18 years arriving in Geneva in 2023

**DOI:** 10.1007/s00431-026-06843-4

**Published:** 2026-03-12

**Authors:** Judith Mühlstein-Barasche, Renato Gualtieri, Noemie Wagner, Catherine Chamay-Weber, Klara Posfay-Barbe, Geraldine Blanchard-Rohner

**Affiliations:** 1 Division of General Pediatrics, Department of Pediatrics, Gynecology and Obstetrics, Faculty of Medicine, Geneva University Hospitalsand , Geneva, Switzerland; 2 Division of Pediatrics, Department of Women-Mother-Child, Lausanne University Hospital and University of Lausanne, Lausanne, Switzerland; 3 Pediatric Infectious Disease Unit, Division of General Pediatrics, Department of Pediatrics, Gynecology and Obstetrics, Geneva University Hospitals and University of Geneva, Geneva, Switzerland; 4 Pediatric Immunology, Vaccinology, and Rheumatology Unit, Division of General Pediatrics, Department of Pediatrics, Gynecology and Obstetrics, Geneva University Hospitals and University of Geneva, 6 Rue Willy-Donzé, Geneva, Switzerland

**Keywords:** Unaccompanied minor asylum seekers (UMAS), Vaccination, Serology, Measles, Varicella, Tetanus, Hepatitis B, Migrant health, Prevention

## Abstract

The recent surge in unaccompanied minor asylum seekers (UMAS) arriving in Switzerland presents significant public health challenges, particularly regarding immunization. Many of these adolescents lack vaccination documentation, requiring catch-up vaccination strategies. This study aimed to evaluate immunity against tetanus, hepatitis B, measles, mumps, rubella and varicella among UMAS upon arrival in Switzerland, to optimize vaccination strategies, and provide protection. This retrospective observational study included 100 UMAS patients followed at Geneva University Hospitals in 2023. Patients received a booster of diphtheria-tetanus-pertussis-inactivated poliovirus vaccine (dTPa-IPV) at the first visit at HUG, followed by serology testing one month later during the second visit. Immunity was defined based on established antibody thresholds. Data on demographics, vaccination history since arrival in Switzerland, and serology for tetanus, hepatitis B, measles, rubella, mumps, and varicella were collected from medical records. Among the 100 UMAS included, 83% originated from Afghanistan, 6% from Burundi, 4% from Somalia, 2% from Eritrea, and the remainder from other countries. The median age was 16 years, and 94% were male. Serological testing on arrival showed that 83% were immune to varicella, whereas immunity to measles was lower at 66%. Immunity rates were higher for mumps (90%) and rubella (89%). Only 22% had protective anti-HBs levels. After administration of a single dTPa-IPV booster, 89% achieved protective tetanus IgG concentrations at one-month follow-up.

*Conclusion*: A substantial proportion of UMAS lacked immunity to measles, hepatitis B, and, to a lesser extent, tetanus and varicella, underscoring the need for systematic catch-up vaccination. Based on these findings, a pragmatic two-visit strategy appears optimal: **Visit 1:** administer MMR (or MMRV), Tdap-IPV, and hepatitis B vaccines to all UMAS; **Visit 2** (after 1 month): repeat MMR (or MMRV) and perform tetanus and hepatitis B serology to guide additional doses. This streamlined approach maximizes early protection while remaining feasible within high-income healthcare settings.

**Supplementary Information:**

The online version contains supplementary material available at https://doi.org/10.1007/s00431-026-06843-4.

## Introduction

The number of unaccompanied minor asylum seekers (UMAS) in Europe has markedly increased since 2021. In both 2022 and 2023, annual applications stabilised around 40,000–42,000, representing approximately 4% of all asylum requests in EU + countries. The main countries of origin remain Afghanistan and Syria, with rising numbers from Somalia, Bangladesh, Venezuela, and Turkey [1]. In Switzerland, the State Secretariat for Migration reported an increase in UMAS applications from 898 in 2021 to 3,871 in 2023; among these, 2,246 were 16- to 17-year-old males from Afghanistan [2]. Many arrive after long and difficult migration journeys, frequently without vaccination documentation, making systematic catch-up immunisation essential.

Migrants and refugees are at increased risk of incomplete vaccination and susceptibility to vaccine-preventable diseases (VPDs), due to interrupted healthcare access, and conflict. Several systematic reviews have confirmed that migrant populations in Europe show lower seroprotection rates against major VPDs compared with host populations [3–5]. In addition, outbreaks of measles, diphtheria, and varicella among asylum seekers and refugees have been reported in recent years [6–8]. Despite this, few studies have simultaneously assessed immunity to multiple VPDs among UMAS, and data in Switzerland remain scarce.

Hepatitis B is one of the most prevalent infections globally, with around 30% of the population having been infected [9]. Migrants from high-endemicity regions show particularly high infection and carriage rates, with a pooled prevalence of approximately 7% on arrival in Europe [3, 10]. Early screening and vaccination are therefore critical to prevent chronic infection and its sequelae. Likewise, suboptimal immunity to tetanus, diphtheria, and poliomyelitis has been documented among adolescent migrants in several european cohorts, reflecting interrupted primary immunisation series [4, 5]. These gaps highlight the need for systematic serological assessment and catch-up vaccination at entry.

Measles continues to represent a global health concern, with 10.3 million cases reported worldwide in 2023, which is a 20% increase from the previous year [11]. Although Switzerland reports approximately 90% two-dose MMR coverage, this level remains below the 95% threshold required for herd immunity. Similar studies in European reception centres have shown that many adolescent migrants lack protective antibody levels for measles and mumps, supporting targeted vaccination efforts [3, 4, 12, 13]. Varicella immunity also varies widely, with 16% of seronegative adult migrants in Spain [14].

While diphtheria and poliomyelitis are now rare in Western Europe, both diseases have re-emerged in under-immunised migrant groups and conflict zones, emphasising the importance of maintaining protection. Recent literature on the European diphtheria outbreak emphasize the importance of dTaP(IPV) vaccination, given the reported fatalities and ongoing transmission among homeless and socioeconomically disadvantaged populations [15–18]. European guidelines stress that migrant children should receive the same vaccines as resident children, yet national catch-up protocols differ markedly, and there is currently no international consensus on vaccination strategies for UMAS [6].

In Switzerland, UMAS are routinely offered catch-up vaccinations upon arrival—including hepatitis B, MMR, varicella, and dTPa or dTPa-IPV—with subsequent serological testing to assess immunity. Patients with tetanus IgG levels between 500–1000 IU/mL received one additional booster dose after six months, whereas those with post-vaccination levels < 500 IU/mL received two additional doses at 6-month intervals. However, in practice, in some centres such as ours, initial serology for hepatitis B and measles is sometimes performed first, and catch-up vaccinations are then administered according to the serological results. However, evidence on actual seroprotection levels in this group and the long-term effectiveness of these strategies is limited. This study therefore aimed to evaluate seroprotection against six key vaccine-preventable diseases: tetanus, hepatitis B, measles, mumps, rubella and varicella, among unaccompanied minor asylum seekers in Geneva, and to review current recommendations to optimise vaccination practices for this vulnerable population.

## Methods

### Study setting and population

This retrospective observational study was conducted at Geneva University Hospitals (HUG) in Switzerland, focusing on UMAS who had their initial consultation at HUG between January and November 2023. Patients with at least 2 available serology were included for analysis. We collected demographic information, including country of origin, as well as available vaccination history and serology results for tetanus, hepatitis B, measles, rubella, mumps, and varicella. All data was obtained from the patients' electronic medical records.

UMAs underwent an initial assessment in a dedicated consultation service for adolescent migrants. Following this visit, they received a tetanus booster (either Tdap or Tdap-IPV). One month later, they returned for blood sampling to assess serology for tetanus and, when indicated, hepatitis B, varicella, and measles, in order to determine the need for catch-up vaccinations. When sufficient serum was available, rubella and mumps serology was additionally performed for the purpose of this study.

Management was variable and not fully standardized. In 2023, many patients and healthcare providers were reluctant to vaccinate without prior serological testing; consequently, serology was more frequently performed before vaccination compared with previous years. When documentation of prior vaccination since arrival in Geneva from another center was available, serology was systematically performed before administering any additional vaccines.

This study was approved by the Geneva Cantonal Ethics Committee on Human Research on September 4, 2023 (study number: ID 2023–01310). The research complied with the Swiss Human Research Ordinance (HRO), specifically the provisions governing the reuse of personal health-related data for research purposes in the absence of consent as outlined in Article 34 of the *Federal Act on Research involving Human Beings (HRA)*.

### Definition of immunity and vaccination status

All serology tests were analyzed using an enzyme linked immunosorbent assay (ELISA) in the routine laboratories of virology for hepatitis B, rubella, and mumps and in the routine laboratories of vaccinology for tetanus, measles and varicella.

Serologies were considered positive for tetanus if antibody level was above the 500 IU/l threshold for long-term protection. Serologies were considered positive for measles if antibody level was above the 150 IU/l threshold, and for varicella if antibody level was above the 100 IU/l, for rubella if antibody level was above > 10 IU/L, for mumps if specific IgG index > 0.2. For hepatitis B, vaccine seroprotection was considered protective if the anti-HBS IgG was above the 10 IU/l threshold before booster vaccination. It is important to note that these seropositivity thresholds are established in clinical practice and have been utilized in prior studies from our group [19, 20].

### Statistical analysis

Continuous data were summarized as median and interquartile ranges (IQRs) when appropriate, or geometric mean concentrations (GMC) with the 95% CI (for antibodies levels). Categorical data were presented as frequencies and percentages.

Given the descriptive design of our study, a sample size calculation was not performed. A post hoc subgroup analysis restricted to participants from Afghanistan (n = 83) was conducted to explore potential differences in seroprotection compared with patients from other origins. No formal statistical testing was performed given the small size of the non-Afghan subgroup and the non-independence between groups.

All analyses were conducted using *Stata* version 18 (StataCorp LLC, College Station, TX, USA). Figures were generated using *GraphPad Prism* version 10.4.0 for Windows (GraphPad Software, San Diego, CA, USA).

## Results

### Patient characteristics

158 UMAS were seen in the HUG between January 2023 and November 2023. Among them, a total of 100 (63%) had at least 2 serology available. 58 were excluded because they had fewer than two serological assessments. The median age was 16 years and 94 were male (94%). Most patients, 83 (83%), were from Afghanistan. The remaining were from Burundi, Somalia, Eritrea and other countries (see Table 1). On average, they arrived in Geneva 106 days (IQR: 58–139) after their arrival in Switzerland, and on average, they were seen by a doctor 28 days after their arrival in Geneva (IQR 16–42). For all UMAS, no reliable vaccination records or history of prior VZV infection were available; only vaccinations administered in Switzerland during the migratory journey could be documented. Consequently, very few patients had received vaccinations before their first evaluation at HUG.

**Table 1:** Demographics

Age, years, median [IQR]	16 [16, 17]
Gender: Male, n (%)	94 (94)
Country of Origin:	
Afghanistan, n (%)	83 (83)
Burundi, n (%)	6 (6)
Somalia, n (%)	4 (4)
Eritrea, n (%)	2 (2)
Other Countries, n (%)	5 (5)
Time to First Consultation since their arrival in Geneva, days, median [IQR]	28 [16–42]
Time to Arrival in Geneva since their arrival in Switzerland, days, median [IQR]	106 [58–139]

### Serological status on arrival in Switzerland

**Fig. 1:**
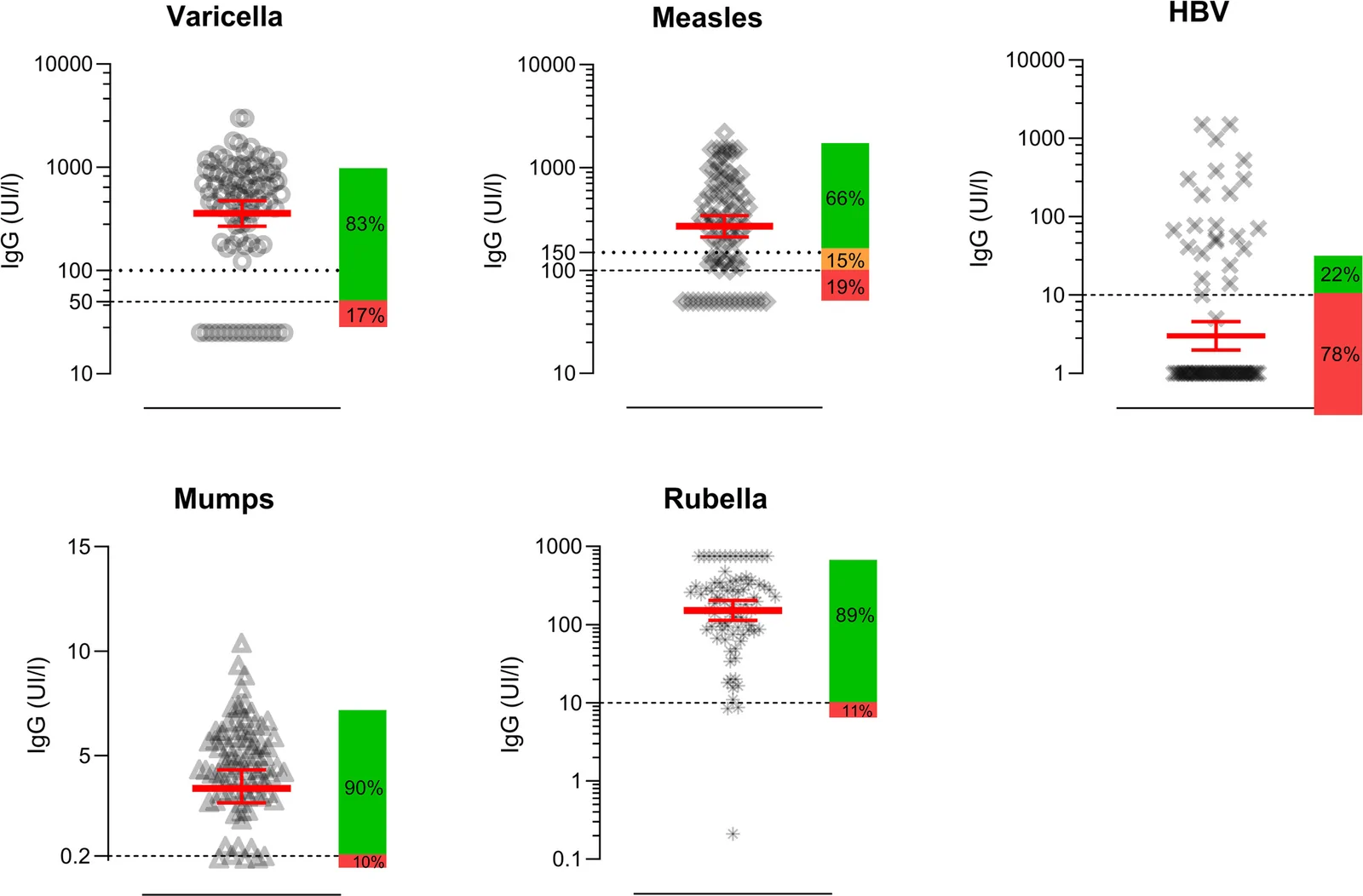
Antigen specific IgG levels of the unaccompanied minor asylum seekers (UMAS) against vaccine-preventable diseases at arrival before any know vaccination. Each symbol represents an individual patient's serological value. The superior dotted line marks the long-term immunity threshold, while the inferior dotted line marks the short-term immunity threshold, according to the disease type. The red line represents the geometric mean concentration with 95% confidence intervals. The histogram represents the percentage of UMAS with protective immunity (in green), or unprotective vaccine immunity (in red), or vaccine serology correlating with short-term protection (in orange)

The vaccine serology profiles of patients upon their arrival in Switzerland, prior to any known vaccinations is summarized in Table 2 and Fig. 1.

**Table 2:** Vaccine seroprotection at the first visit (before any known vaccination)

	Tested, N(%)	Protected, N(%)	Serology, GMC [95%]
Varicella	89 (89)	74/89 (83)	357 [269–476], UI/l
Measles	87 (87)	57/87 (66)	269 [212–342], UI/l
Mumps	83 (83)	75/83 (90)	3.4 [2.7–4.3], index
Rubella	83 (83)	74/83 (89)	152 [113–205], UI/l
HBV	98 (98)	22/98 (22)	3.0 [2.0–4.6], UI/l

Only vaccinations administered in Switzerland during the migratory journey could be documented. Consequently, very few patients had received vaccinations before their first evaluation at HUG. Based on the available records, 9 patients received a tetanus-containing vaccine, 6 received MMR, and 6 received a VZV vaccine prior to baseline serological assessment; these patients as well as those receiving vaccinations at arrival at HUG were therefore excluded from the baseline serology analysis.

Concerning chickenpox, 74/89 (83%) were immune to varicella. Regarding Measles, Mumps, and Rubella (MMR), among 87 patients tested for measles, 57 (66%) were immune. Immunity rates for mumps and rubella were higher, with 90% (75/83) and 89% (74/83) of patients, respectively, showing immunity. Notably, no patient tested positive for measles alone. Among the 82 patients with serologies for measles, mumps, and rubella, 44 were immune to all three diseases.

Complete hepatitis B serological testing was performed in all UMAS, including HBsAg, anti-HBs, anti-HBc, and anti-HBe, allowing differentiation between vaccine-induced immunity, past infection, and active disease.

Among the 25 UMAS with positive hepatitis B serology, 15 (60%) showed a vaccination profile (anti-HBs positive, anti-HBc negative), including three with borderline anti-HBs titers. Seven patients (28%) had evidence of resolved hepatitis B infection (anti-HBs and anti-HBc positive). Three patients (12%) displayed indeterminate serological profiles, characterized by isolated or borderline anti-HBc and/or anti-HBe positivity; these patterns were considered most consistent with past resolved infection and were excluded from serological analyses to avoid misclassification. Overall, 22% (22/98) of patients tested positive with a mean IgG level of anti-HBs antibodies of 3 IU/l. One patient (4%) had evidence of active hepatitis B infection, with HBsAg positivity, and was referred for specialist hepatology follow-up.

This comprehensive serological approach ensured appropriate clinical management and accurate interpretation of hepatitis B immune status in this population.

**Fig. 2:**
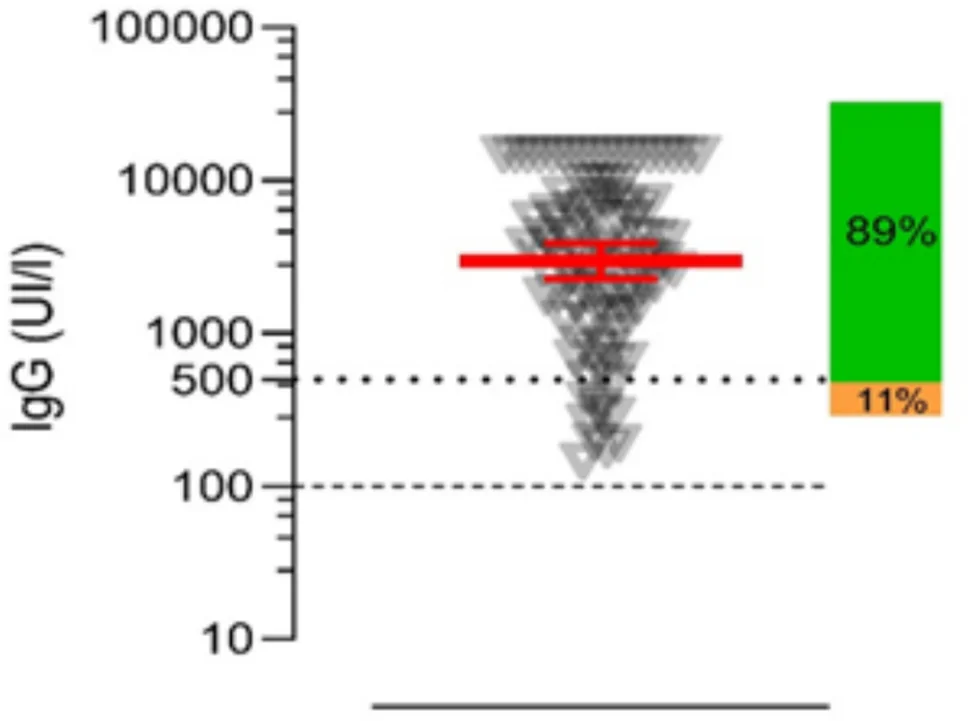
Tetanus specific IgG levels of UMAS one month following booster vaccination with Tdap-IPV. The superior dotted line marks the long-term immunity threshold, while the inferior dotted line marks the short-term immunity threshold, according to the disease type. The red line represents the geometric mean concentration with 95% confidence intervals. The histogram represents the percentage of UMAS with protective immunity (in green), or vaccine serology correlating with short-term protection (in orange)

The UMAs received tetanus-containing vaccines during their migratory journey in Switzerland or at their arrival at HUG, and tetanus IgG titers were therefore measured in these patients following the booster. Among them, those with tetanus IgG levels < 500 IU/mL received two additional doses of diphtheria–tetanus vaccine at 6-month intervals, whereas those with titers between 500 and 1000 IU/mL received one additional booster dose 6 months later. It should be noted that follow-up post-vaccination serology was usually not done. A total of 91 patients received a tetanus booster at HUG. Among them, 81/91 patients (89%) had an IgG level greater than 500 IU/l, with a geometric mean concentration (GMC) of 2979 IU/l (see Fig. 2). In the post hoc subgroup analysis restricted to Afghan participants, seroprotection levels were comparable to those observed in the overall cohort (Supplementary Table 1).

## Discussion

The serological results observed among UMAS in our cohort highlight substantial immunity gaps to several VPDs, notably tetanus, varicella, measles, and hepatitis B. Similar findings have been reported in other European and high-income settings, suggesting that incomplete immunisation and undocumented vaccination histories remain major challenges in this population [1, 3–5]. These results reinforce the importance of systematic catch-up vaccination and serological follow-up in UMAS upon arrival.

Consistent with prior studies in migrant populations across Europe, our findings showed that approximately one-third of patients lacked protective measles antibodies. This aligns with previous studies; Among a large cohort of refugees in Germany, the study reported 22% measles seronegativity in some origin subgroups [21]. Another meta-analytic data reported ~ 13–17% measles seronegativity among migrants [22]. Given the ongoing circulation of measles and its outbreak potential in refugee reception centres, a universal vaccination strategy remains warranted. As recommended by the World Health Organization (WHO) and the European Centre for Disease Prevention and Control (ECDC), all individuals without documented evidence of two MMR doses should receive two doses at least four weeks apart, regardless of serological status [6, 23]. While post-vaccination serology can provide additional information, it is not routinely advised for assessing measles immunity in healthy individuals, as antibody titres may decline without indicating a loss of immune memory. Therefore, our recommendation for administering two MMR doses aligns with international guidelines rather than solely with local practice. Moreover, studies indicate that even a third MMR dose in individuals with waning antibody levels typically results in only transient increases in titres [24]. Consequently, repeated serology-driven revaccination is unlikely to confer lasting benefit. Ensuring that all UMAS complete the full two-dose schedule—ideally at the first clinical encounter—represents the most evidence-based and pragmatic approach.

In our cohort, 83% of participants were immune to varicella. This is somewhat lower than seroprevalence reported in several refugee/newly-arrived migrant cohorts (typically ≥ 90% in Germany and other series), although localized vulnerability can occur, as reported in migrants from West and East Africa in an asylum center in Italy and among Sudanese refugees in the Calais camp [21, 25, 26]. The ECDC recommends vaccination for individuals without evidence of prior infection or documented vaccination [6]. Serological testing before vaccination may be useful in adolescents and adults from tropical countries, where a higher proportion may remain susceptible.

Cost considerations for varicella-zoster virus (VZV) management were informed by a comparison between serological testing and vaccination using standard Swiss healthcare tariffs. The estimated cost of complete VZV vaccination, including two doses and nursing administration, was CHF 135.30, whereas VZV serological testing, including laboratory analysis and blood sampling, cost approximately CHF 63.25.

In this population of unaccompanied minor asylum seekers (UMAS), serological testing was therefore selectively performed to avoid unnecessary vaccination in individuals with pre-existing immunity, given the high probability of prior natural infection among adolescents from endemic regions. This strategy remained consistent with Swiss vaccination recommendations for individuals without documented immunity.

With respect to tetanus, our findings confirm high seroconversion (89%) after a single booster dose of a tetanus-containing vaccine. This aligns with previous data showing that a single dT booster restores protective antibody levels in most migrant adolescents[27, 28]. Nevertheless, post-vaccination serology remains valuable to confirm long-term protection, as some individuals may respond suboptimally, especially when basic immunisation series were incomplete [23]. In cases where post-booster titres remain below 500 mIU/mL, an additional dT dose is justified to ensure sustained protection. The inclusion of diphtheria and poliovirus antigens within the same booster (Tdap-IPV) allows harmonisation with national schedules and optimises coverage with minimal additional injections.

Hepatitis B immunity was also incomplete in our cohort, with only 22% showing protective anti-HBs levels prior to vaccination. This is broadly consistent with reports from European migrant and refugee cohorts, where pre-vaccination anti-HBs immunity is often low — for instance, one study from Catalonia found only ~ 16% of migrants had protective anti-HBs on arrival, and a global meta-analysis estimated the pooled prior immunity at ~ 40% [10, 29]. Our approach—initiating vaccination and guiding completion based on post-vaccine serology aligns with WHO and Swiss Federal Office of Public Health recommendations for high-risk or undocumented individuals [23, 30]. Given that antibody levels may decline over time despite persistent immune memory, post-vaccination serology should be interpreted cautiously. For practical purposes, a single vaccine dose followed by antibody testing can guide whether additional doses (up to three in total) are required, thereby avoiding both under- and over-vaccination.

The findings from our study must also be interpreted in light of the population composition: approximately three-quarters of participants originated from Afghanistan, where recent estimates indicate that only 37% of children aged 12–23 months were fully vaccinated in 2019 [31]. While low immunization coverage is well documented in settings like Somalia, where routine vaccine coverage has sometimes been estimated at just 30–40% in certain studies [32], the situation is more complex in Eritrea, which historically has had relatively high (≈ 80–95%) reported childhood vaccination coverage [33]. Therefore, our cohort likely reflects a population at high risk of incomplete immunisation, representative of the broader UMAS population arriving in Western Europe.

Currently, many UMAs originate from Syria. Unfortunately, Syrian UMAs were not represented in our cohort, and therefore we cannot draw conclusions specific to this population based on our data. However, published reports and surveillance data indicate that vaccination coverage in Syria has markedly declined since the onset of the conflict, with significant regional heterogeneity and repeated disruptions of routine immunization programs. Consequently, we would expect a similarly high proportion of non-immune individuals among Syrian UMAs, particularly for measles and varicella, as has been observed in other migrant populations from countries with disrupted healthcare systems. This underscores the importance of systematic assessment and catch-up vaccination strategies for all UMAs, regardless of country of origin.

These results underscore the need for harmonized European guidelines on catch-up vaccination in UMAS, as current recommendations vary between countries. Standardized, evidence-based protocols for newly arrived minors would improve both equity and efficiency. Practical feasibility is a key consideration: many UMAS face barriers that can prevent complete vaccination, including frequent relocations between reception centres or foster homes, competing obligations such as school or legal appointments, and limited availability of healthcare staff to track and follow up with each child.

A pragmatic two-visit outpatient schedule could be:

- Visit 1: Administer MMR (or MMRV), Tdap-IPV, and hepatitis B vaccines to all UMAS.
- Visit 2: Repeat MMR (or MMRV) and perform serology for tetanus and hepatitis B to guide additional doses if needed.

This approach aligns with ECDC guidance recommending early administration of priority vaccines while minimizing missed opportunities [6]. Evidence shows that combining multiple vaccines in one visit, structured follow-up, and culturally and linguistically adapted information and support improves vaccine uptake among UMAS [34].

Although this is a single-center study with a predominantly Afghan cohort, our findings provide real-world data on immunity gaps in UMAS. In line with WHO, ECDC, and Swiss recommendations, we support administering two MMR doses regardless of initial serology, targeted varicella vaccination for non-immune individuals, including those with unknown vaccination or infection history, Tdap-IPV and hepatitis B boosters with post-vaccination serology follow-up, and additional vaccination guided by serology. Integrating these strategies into broader European public health initiatives is essential to ensure equitable protection for all young migrants.

## Supplementary Information

Below is the link to the electronic supplementary material.

ESM 1 DOCX (33.0 KB)

## Data Availability

No datasets were generated or analysed during the current study.

## Abbreviations

UMAS: Unaccompanied minor asylum seeker
HUG: University Hospitals of Geneva
MMR: Measles, Mumps and Rubella vaccine
VPD: Vaccine-preventable diseases
