# Supplementary material for: Evaluation of vaccine immunity against tetanus, hepatitis B, measles, mumps, rubella and varicella, in migrant adolescents aged 13 to 18 years arriving in Geneva in 2023
Source: Eur J Pediatr. 2026 Mar 12;185(4):177. doi: 10.1007/s00431-026-06843-4 (PMC13676514; doi:10.1007/s00431-026-06843-4)
Supplement: Supplementary file 1 [file 431_2026_6843_MOESM1_ESM.docx]

**Supplementary Table 1**: Vaccine seroprotection at the first visit (before any known vaccination) comparing all patients and Afghans only.

|  | All (N=100) | | | Afghan subgroup (N=83) | | |
| --- | --- | --- | --- | --- | --- | --- |
|  | **Tested,**  **N(%)** | **Protected,**  **N(%)** | **Serology,**  **GMC [95% CI]** | **Tested,**  **N(%)** | **Protected,**  **N(%)** | **Serology,**  **GMC [95% CI]** |
| Varicella | 89 (89) | 74/89 (83) | 357.5 [268.6–475.8], UI/l | 72 (87) | 60/72 (83) | 356.9 [259.5–490.9], UI/l |
| Measles | 87 (87) | 57/87 (66) | 269.0 [211.5–342.3], UI/l | 71 (86) | 49/71 (69) | 280.9 [214.9–367.1], UI/l |
| Mumps | 83 (83) | 75/83 (90) | 3.4 [2.7–4.3], Index | 69 (83) | 63/69 (91) | 3.7 [2.8–4.7], Index |
| Rubella | 83 (83) | 74/83 (89) | 152.1 [113.0–204.7], UI/l | 69 (83) | 62/69 (90) | 152.5 [110.1–211.1], UI/l |
| HBV | 98 (98) | 22/98 (22) | 3.0 [2.0–4.6], UI/l | 82 (99) | 20/82 (24) | 3.3 [2.1–5.2], UI/l |
| Tetanus | 9 (9) | 9/9 (100) | 508.4 [115.5–2236.8], UI/l | 7 (8) | 7/7 (100) | 374.6 [63.0–2225.5], UI/l |
